# Global Transmission Dynamics of Measles in the Measles Elimination Era

**DOI:** 10.3390/v9040082

**Published:** 2017-04-16

**Authors:** Yuki Furuse, Hitoshi Oshitani

**Affiliations:** Tohoku University Graduate School of Medicine, 980-8575 Sendai, Japan; oshitanih@med.tohoku.ac.jp

**Keywords:** measles, transmission, endemic, outbreak, globalization, phylogeography, elimination

## Abstract

Although there have been many epidemiological reports of the inter-country transmission of measles, systematic analysis of the global transmission dynamics of the measles virus (MV) is limited. In this study, we applied phylogeographic analysis to characterize the global transmission dynamics of the MV using large-scale genetic sequence data (obtained for 7456 sequences) from 115 countries between 1954 and 2015. These analyses reveal the spatial and temporal characteristics of global transmission of the virus, especially in Australia, China, India, Japan, the UK, and the USA in the period since 1990. The transmission is frequently observed, not only within the same region but also among distant and frequently visited areas. Frequencies of export from measles-endemic countries, such as China, India, and Japan are high but decreasing, while the frequencies from countries where measles is no longer endemic, such as Australia, the UK, and the USA, are low but slightly increasing. The world is heading toward measles eradication, but the disease is still transmitted regionally and globally. Our analysis reveals that countries wherein measles is endemic and those having eliminated the disease (apart from occasional outbreaks) both remain a source of global transmission in this measles elimination era. It is therefore crucial to maintain vigilance in efforts to monitor and eradicate measles globally.

## 1. Introduction

The measles virus (MV) is responsible for a highly contagious febrile disease characterized by a rash; it remains one of the leading causes of death among young children worldwide despite the availability of a safe and effective vaccine. According to the World Health Organization (WHO), over 130,000 children died worldwide from the disease in 2015 [1]. MV has a single serotype; however, it can be classified into various genotypes based on their genetic sequences. In countries where measles remains endemic, one or a few specific genotypes circulate geographically, e.g., genotype H1 in China and genotype B3 in Africa [2,3]. Since the development of the vaccine, many countries have eliminated endemic measles [4], but the risk of the introduction of MV from other countries remains prevalent. The genotype of the newly introduced MV often differs from the indigenous genotype. Shifts in the genotypes of endemic strains have also been documented [5,6,7]. Therefore, it has become difficult to decipher the global transmission dynamics of the disease based solely on virus genotypes.

Recently, the Bayesian inference technique has been developed for phylogeographic analyses of geographic information through the evolutionary pathway. [8]. This technique can elucidate the spatial and temporal transmission dynamics of microbes, as has been done in studies of avian influenza virus [8], human immunodeficiency virus [9], hepatitis C virus [10], and *Salmonella typhimurium* [11]. Although there have been many epidemiological reports about inter-country transmission of measles [12,13,14,15], systematic analysis of the global transmission dynamics of MV has been limited.

In this study, we employed phylogeographic analysis to characterize the global transmission dynamics of MV using large-scale genetic sequence data (a total of 7456 sequences). This analysis has revealed the spatial and temporal characteristics of global transmission of the virus, especially in Australia, China, India, Japan, the UK, and the USA since 1990, for which an abundance of sequence data is available. The aim of the present study was to provide a comprehensive account of the temporal and spatial transmission dynamics of measles worldwide.

## 2. Materials and Methods

### 2.1. Sequence Data

Genetic sequence data of the *N* (nucleoprotein) gene of MV, including information on isolation country and year, were obtained from the Measles Nucleotide Surveillance Database [5,16]. The *N* gene was selected for analyses because it is the most commonly sequenced, registered, and used gene for genotype classification of MV. Vaccine strains, laboratory strains, and isolates from subacute sclerosing panencephalitis (SSPE) patients were excluded from further analyses.

Sequence data from a total of 7456 strains from 115 countries for the period from 1954 to 2015 were aligned. A 450-nucleotide region of the *N* gene is the sequence most commonly reported in the Measles Nucleotide Surveillance Database for nomenclature of MV genotypes [17]. Since some data include only a part of the sequence of this region, a subset of 382 nucleotides in the 450-nucleotide region was identified as the sub-region sequenced in most of the sequence data and was therefore selected for further analyses. We confirmed that the topologies of the phylogenetic trees with the partial 382-nucleotide sequence and the 450-nucleotide sequence are identical. A list of the accession numbers of the sequences analyzed in the study can be found in Data S1.

### 2.2. Phylogeographic Analysis

Estimation of evolution rates, divergence times, and phylogeography of the virus was performed using the BEAST version 1.8.2 that uses Bayesian Markov chain Monte Carlo (MCMC) algorithm [18]. A relaxed uncorrelated lognormal clock, which allows the evolutionary rates to change among the branches of the tree, was used, according to the General Time Reversible (GTR) substitution model. The geographic locations by region and country of interest regarding ancestral nodes were estimated using the discrete geospatial model under Bayesian stochastic search variable selection implemented in BEAST [8]. One hundred million iterations were performed in the MCMC analysis and were sampled every 100,000 steps with a 10% burn-in of MCMC generations. Transmission of MV was defined in terms of the transition of the estimated location site at the nodes and sampling location site at the tips in the maximum clade credibility phylogenetic tree. Transmissions to and from a country of interest were identified based on phylogenetic trees constructed for each country. Transmissions between nodes (or between a node and a taxon) for which both posterior probabilities are equal to or greater than 0.85 were regarded as statistically credible. Maximum clade credibility trees were created and annotated using TreeAnnotator version 1.8.2 and viewed in FigTree version 1.4.2.

The MV source index was calculated to describe the magnitude of the relationship between the numbers of import and export as follows:

$$\mathrm{MV}\;\mathrm{source}\;\mathrm{index}=\;\frac{\mathrm{Frequency}\;\mathrm{of}\;\mathrm{origin}\;-\;\mathrm{Frequency}\;\mathrm{of}\;\mathrm{destination}}{\mathrm{Frequency}\;\mathrm{of}\;\mathrm{origin}\;+\;\mathrm{Frequence}\;\mathrm{of}\;\mathrm{destination}}$$

when the value is greater than 0, a country can be assumed to be a source of MV transmission; when the value is less than 0, a country can be assumed to be a recipient of introduced MV. The indices were smoothed using a Kernel average smoother (with an 8-year window).

## 3. Results

### 3.1. Phylogeography of Measles Virus

Sequence data of 7456 strains of MV with epidemiological information including isolation country and year were obtained from the Measles Nucleotide Surveillance [5,19]. The dataset contains sequence data on MV strains identified from 115 countries between 1954 and 2015. The number of sequences analyzed is listed by time and the WHO regional level [20], namely the African region, region of the Americas, Eastern Mediterranean region, European region, South-East Asia region, and Western Pacific region, as shown in Table 1. According to our phylogenetic analysis, the time to the most recent common ancestor (MRCA) of the current MV was 106.6 years (95% credible interval: 72.5–146.3) from 2015; i.e., the MRCA existed around 1908. The fact that this estimation is compatible with previous studies confirms the robustness of our dataset and phylogenetic model [21,22].

**Figure 1:**
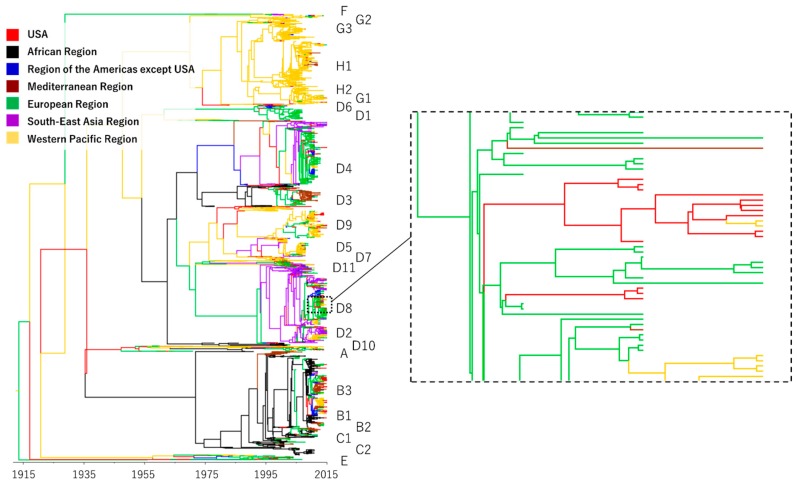
Maximum clade credibility phylogeny for a portion of the N gene of measles virus (MV). Time-scaled phylogenies were inferred for analysis of measles virus transmissions in the USA using a relaxed molecular clock model in a Bayesian MCMC algorithm that incorporates virus sampling dates (year) and sampling location (i.e., the USA or one of the six WHO regions) to estimate phylogenetic trees concurrently. Tip times reflect the year of sampling, and branches are colored according to the most probable location of their descendent nodes or sampling location site of tips. Genotypes are indicated on the right side of the tree. Areas bordered by broken lines are magnified in the right panel.

The phylogenetic trees, with the geographical information at each node and taxon, can be used to infer global transmission of MV through evolutionary history since the early twentieth century. Geographical information regarding MV was analyzed for the six selected countries from where most of the sequences were gathered (Australia, China, India, Japan, the UK, and the USA) and for the WHO regional levels. Figure 1 shows the phylogenetic tree for transmission of MV in the USA. For the analysis, location variates of the African region, region of the Americas except the USA, Eastern Mediterranean region, European region, South-East Asia region, Western Pacific region, and the USA were provided as geographical information. The transitions of colors in the branches of the tree indicate global transmission of the virus. Phylogenetic trees for the other five countries were also constructed; these allow us to identify statistically credible global transmission of MV in those locations. When we analyzed transmission of MV in Japan, for another example, location variates of the African region, region of the Americas (including the USA), Eastern Mediterranean region, European region, South-East Asia region, Western Pacific region except Japan, and Japan were provided as geographical information. Table 2 shows the number of transmissions identified in each country. A few instances of transmission were observed before 1990, but most were not statistically credible and were therefore excluded from further analysis.

### 3.2. Geographical Trend in Measles Transmission

**Figure 2:**
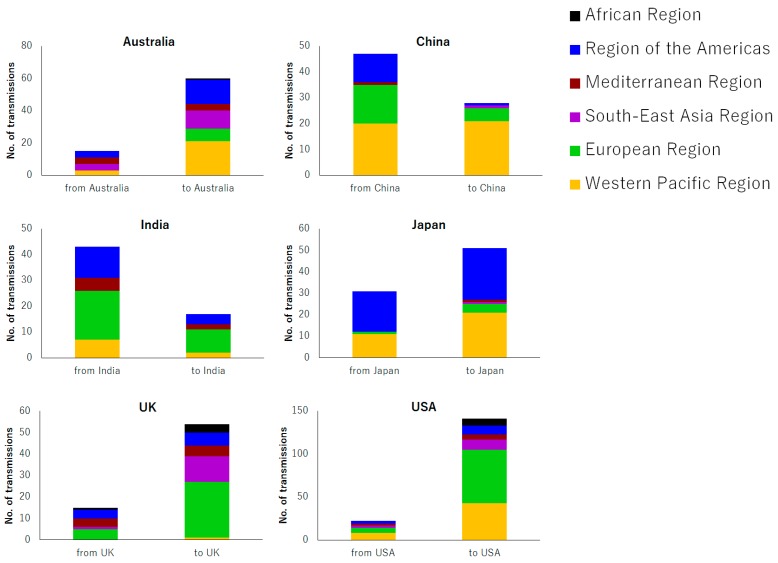
Source and destination of global transmission.

Figure 2 shows the geographic source and destination of MV import and export for the six countries of interest. Transmission was frequently observed within areas geographically proximal to each other, as shown by transmissions between China and the Western Pacific region, between Japan and the Western Pacific region, and between the UK and the European region. In addition, in some cases, frequent transmission was observed between geographically distant areas as well, such as the transmission between India and the European region and that between Japan and the region of the Americas.

Geographical characteristics of statistically credible instances of global transmission of measles virus in six countries are shown. Source and destination of the transmission are described by the WHO regions.

### 3.3. Temporal Trend of Measles Transmission

**Figure 3:**
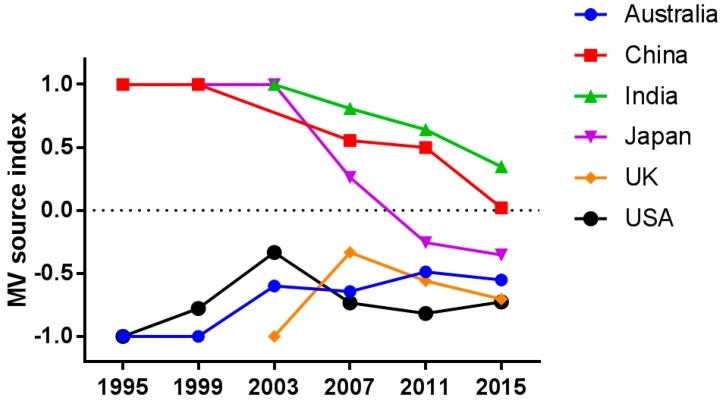
Measles virus (MV) source index. Temporal trend in the MV source index for six countries indicating whether a country of interest is a source or destination of global transmission. The MV source index represents the relative frequency of import and export (see Materials and Methods for detail). Data before 1995 are not shown because few statistically credible instances of transmission were identified before that year.

Because the instances of transmissions have increased, as has the amount of sequence data available (Table 2), the temporal trend in the relationship between the export and import of MV cannot be discussed in terms of the absolute volume of instances of transmission. For this reason, for the six countries, we calculated the MV source index, a value that represents the relative frequency of import and export (see Materials and Methods for detail). The value of the MV source index was greater than zero for a country that was a source of MV transmission (1 being the maximum) and was smaller than zero for a country that was a recipient of foreign MV (−1 being the minimum). As expected, the index was high for countries where indigenous MV still circulates (i.e., China and India) low for countries where local transmission of MV has been interrupted (i.e., Australia, the UK, and the USA; Figure 3).

The index in Japan was greater than zero until 2007 and decreased to below zero thereafter. Once regarded an exporter of MV [23,24], Japan declared in 2015 that it had eliminated measles [25]. Similarly, the indices in China and India are decreasing, although the values remain greater than 0.

It should also be noted that the indices are marginally increasing in Australia, the UK, and the USA, countries where measles is no longer endemic [26,27,28]. Our findings suggest that occasional outbreaks in countries where MV has been eliminated may also become sources for further global transmission of the disease. As can be seen in Figure 2 (right panel), MV that had been introduced from the European region to the USA was then further transmitted from the USA to the Western Pacific region. These findings suggest that countries where measles has been declared to be eliminated represent a new source of global MV transmission.

## 4. Discussion

In this study, we have conducted a systematic phylogeographic analysis using viral genetic sequence data to analyze the global transmission dynamics of MV. Transmission was frequently observed within a given region, as was transmission between distant areas, including that between India and the European region and between Japan and the region of the Americas. Although vast distances separate such areas geographically, the considerable volume of travelers among them has the potential to facilitate inter-regional transmission of MV (Figure 2) [29].

The instance of MV transmission has increased since 1995 in most countries in terms of both import and export (Table 2). The increased detection of inter-country transmission may be due to globalization and the increasing availability of sequence data. In addition, imported strains are more likely to be detected in countries where the elimination of endemic strains has already been achieved. Imported strains of MV may not be detected when endemic strains are still circulating. It is notable that the relative frequency of the export of MV from measles-endemic countries is high but decreasing (Figure 3). Measures such as increasing vaccination coverage and enhanced surveillance for the disease are likely eliminating measles in these countries, which resulted in relatively less frequency of export from those countries. Conversely, the relative frequency of MV export from countries where measles is no longer endemic is low but slightly increasing (Figure 3). Such countries continue to be affected by occasional outbreaks caused by imported MV [6,30,31,32]. Moreover, the risk of MV import into such countries can increase when vaccination coverage decreases. Indeed, incomplete vaccination in countries from which measles had been eliminated has already resulted in serious outbreaks of the disease [33,34,35,36,37], and such outbreaks have the potential to spread the virus to other parts of the world, as has in fact recently been reported [38,39].

Other published studies monitoring the global distribution of MV genotypes have also discussed global transmission of the virus [5,19]. The introduction of new genotypes indicates import of the virus, but clear geographical clustering of MV genotypes no longer occurs in this measles elimination era [5]. When MV of a genotype endemic within a region causes an outbreak in a distant area where it is not endemic, the virus can be further transmitted from the outbreak location to another area, as illustrated in Figure 1. Identification of the genotype is thus insufficient to determine the source of an MV isolate and could foster misunderstanding regarding the actual paths of transmission. We have attempted to tackle this issue in the present study by using phylogeographic analyses to infer places of origin and destinations for each instance of transmission.

It should be noted, however, that the genetic data on MV are insufficient to account for the entire global transmission picture since data from many countries are still missing. We therefore combined data from different countries into regions and focused on global transmission in six countries for which data were readily available. These six are all high- and middle-income countries. Unfortunately, we lack data from low-income countries. There is also a concern regarding bias in sampling. For example, the viruses in Africa could be underrepresented, although we regard that the number and distribution of samples from African region (875 sequence data from 33 countries) are not critically underrepresented for our analyses. A further concern is that the sequence data for the virus are insufficient in terms of temporal distribution. Unfortunately, we detected too few instances of transmission before 1990 to allow for analysis of temporal change in global transmission of the virus prior to that date, as interesting as such an analysis would be.

Another limitation of this study is the short sequence (382 nucleotides) used in our analyses, which represents only a part of the *N* gene recommended for genotype classification (450 nucleotides). It is expected that future surveillance will analyze sequence data using a longer region, owing to recent advances in sequencing technology, thus conferring increased validity on this approach.

The world is heading toward measles eradication, but transmission of the virus still occurs regionally and globally. Even countries have that have effectively eliminated measles can experience occasional outbreaks and consequently become sources of global transmission. Vigilance and enhanced testing are therefore necessary to eradicate the disease worldwide. This vigilance must extend to areas in which measles has been declared eliminated, since they clearly still play a role in transmission of the disease. Above all, we need more epidemiological information regarding measles and more MV sequence data from all over the world.

## Figures and Tables

**Table 1:** Number of sequences by region analyzed in this study.

	African Region	Region of the Americas	Mediterranean Region	European Region	South-East Asia Region	Western Pacific Region	Total
Before 1955	0	1	0	0	0	0	1
1956–1965	0	2	0	5	0	2	9
1966–1975	0	2	0	4	0	5	11
1976–1985	12	5	0	6	0	15	38
1986–1995	25	19	0	15	1	46	106
1996–2005	292	144	140	275	98	687	1636
2006–2015	546	803	522	1495	456	1793	5655
Total	875	976	662	1800	595	2548	7456

**Table 2:** Number of sequences and transmissions by country.

	Australia			China			India			Japan			UK			USA		
	Sequence	Transmission ^a^		Sequence	Transmission ^a^		Sequence	Transmission ^a^		Sequence	Transmission ^a^		Sequence	Transmission ^a^		Sequence	Transmission ^a^	
		from Australia	to Australia		from China	to China		from India	to India		from Japan	to Japan		from UK	to UK		from USA	to USA
Before 1955	0	0 (0)	0 (0)	0	0 (0)	0 (0)	0	0 (0)	0 (0)	0	0 (0)	0 (0)	0	0 (1)	0 (1)	1	0 (1)	0 (2)
1956–1965	0	0 (1)	0 (1)	2	0 (0)	0 (1)	0	0 (0)	0 (0)	0	0 (0)	0 (1)	1	0 (0)	0 (0)	2	0 (3)	0 (2)
1966–1975	4	0 (1)	0 (1)	0	0 (0)	0 (1)	0	0 (0)	0 (0)	1	0 (2)	0 (1)	2	0 (0)	1 (3)	2	0 (2)	0 (2)
1976–1985	10	0 (1)	0 (0)	0	0 (1)	0 (0)	0	0 (0)	0 (0)	5	0 (0)	0 (1)	2	0 (1)	0 (0)	4	0 (2)	0 (3)
1986–1995	13	0 (1)	1 (4)	31	1 (3)	0 (4)	0	0 (0)	0 (3)	0	0 (0)	0 (2)	2	0 (2)	0 (0)	15	0 (3)	2 (9)
1996–2005	54	4 (15)	19 (8)	419	14 (21)	3 (8)	71	5 (8)	2 (9)	80	8 (14)	3 (11)	16	4 (7)	6 (13)	98	6 (26)	27 (56)
2006–2015	276	11 (20)	40 (34)	842	32 (42)	25 (34)	446	38 (58)	15 (32)	408	23 (27)	48 (65)	141	11 (28)	47 (67)	456	16 (39)	112 (140)
Total	357	15 (39)	60 (48)	1294	47 (67)	28 (48)	517	43 (66)	17 (44)	494	31 (43)	51 (81)	164	15 (39)	54 (84)	578	22 (76)	141 (214)

^a^ A transmission was defined as a transition of estimated location site at nodes and sampling location site at tips in a maximum clade credibility-phylogenetic tree. Transmissions to and from a country of interest were identified from phylogenetic trees constructed for each country. Values indicate the number of statistically credible transmissions with a posterior probability equal to or greater than 0.85, and values in parenthesis indicate the number of all transmissions irrespective of posterior probability.

## Supplementary Materials

Data S1: list of accession numbers of sequence analyzed in the study.
